# Correlation Between Prognostic Indicators, HPV Status, and Cancer Stem Cell Markers in a Population of Human Tongue Carcinomas

**DOI:** 10.1111/jop.70175

**Published:** 2026-08-16

**Authors:** Adriely Primo da Silva, Beatriz Gomes Carreira Sartori, Carolina de Barros Gigliotti Vendramini, Adauto José Ferreira Nunes, Maria Renata Sales Nogueira

**Affiliations:** ^1^ Universidade Estadual Paulista (UNESP) Bauru SP Brazil; ^2^ Instituto Lauro de Souza Lima (ILSL) Bauru SP Brazil; ^3^ Hospital Amaral Carvalho (HAC) Jaú SP Brazil

**Keywords:** ALDH1, cancer stem cells, CD44, HPV, oral tongue cancer

## Abstract

**Background:**

Oral squamous cell carcinoma (OSCC) is a significant public health issue. While tobacco and alcohol are established risk factors, the role of human papillomavirus (HPV) in oral carcinogenesis remains debated. Cancer stem cells (CSCs) are implicated in tumor aggressiveness and therapy resistance. This study investigated the correlation between HPV infection, CSC marker expression (CD44, CD98, ALDH1), and clinicopathological parameters in tongue SCCs.

**Methods:**

A retrospective cohort of 1047 OSCC cases (2013–2018) was reviewed. From 452 tongue SCCs, 83 formalin‐fixed paraffin‐embedded specimens were randomly selected. HPV DNA detection was performed using qPCR with generic and type‐specific primers (HPV 6, 11, 16, 18). Immunohistochemical expression of CD44, CD98, and ALDH1 was evaluated and quantified using digital image analysis (IHC Profiler). Statistical analyses included Kaplan–Meier survival curves, Kruskal‐Wallis, Chi‐square, and Spearman correlation tests.

**Results:**

HPV DNA was detected in 2/83 cases (2.4%): one HPV16‐positive and one with co‐infection (HPV6/18). CD44 expression was ubiquitously high, with no significant difference between ‐High and ‐Positive categories (*p* > 0.9999), but both were significantly higher than ‐Low and ‐Negative scores (*p* < 0.0001). ALDH1 exhibited a focal staining pattern. The ALDH1‐High score was significantly less frequent than ‐Positive and ‐Low scores (*p* < 0.01) and showed a significant positive correlation with both advanced clinical stage (Spearman r: 0.5062; *p* = 0.0007) and higher tumor grade (Spearman r: 0.3352; *p* = 0.0322). CD98 was not detected. An inverse correlation was observed between HPV and clinical staging (Spearman r: −0.2377; *p* = 0.0305); however, due to the low number of HPV‐positive cases, this finding must be interpreted with caution.

**Conclusion:**

Our findings suggest a distinct CSC expression profile in this cohort, characterized by ubiquitous CD44 expression and a rare but highly aggressive ALDH1‐High subpopulation, concomitant with low HPV detection. The strong positive correlation of ALDH1‐High score with advanced stage and grade highlights its potential role as a key driver of tumor aggression, warranting further investigation as a prognostic marker.

## 1 Introduction

### 1.1 The Global and Local Burden of Oral Tongue Cancer

Oral cancer represents a significant global health issue, with 2024 estimates indicating 452,205 new cases and 194,108 deaths annually for lip and oral cavity cancers worldwide [1]. The majority of these malignancies are squamous cell carcinomas, which frequently affect the tongue [2] alongside other anatomical sites such as the lips, floor of the mouth, buccal mucosa, hard palate, and retromolar trigone [3]. This global burden has a particularly high impact in specific developing regions [4], with high incidence and mortality rates observed in South and Southeast Asia [3]. The significant public health impact of oral cancer is starkly illustrated in Brazil, where recent epidemiological data from the Brazilian National Cancer Institute [5] estimate 22,890 new head and neck cancer cases annually for the 2023‐2025 period [6]. Among head and neck cancer subtypes, oral cavity cancer is the most common and currently ranks as the fifth most frequent cancer among men in Brazil. This high incidence, paired with 11,334 head and neck cancer deaths recorded nationwide in 2023, underscores its status as a critical health priority in the country [6].

### 1.2 Multifactorial Carcinogenesis: From Traditional Risks to Viral Etiology

The development of OSCC is characterized by its multifactorial etiology. Well‐established behavioral risk factors, notably tobacco smoke and alcohol consumption, demonstrate a potent synergistic effect that confers up to a 30‐fold increased risk [5]. The carcinogenic potential of tobacco operates through multiple mechanisms, including direct DNA damage from thousands of chemical compounds, induction of oxidative stress, and epigenetic modifications [7]. Similarly, ethanol metabolism generates acetaldehyde, which exerts genotoxic effects and impairs DNA repair pathways. Beyond these exogenous factors, infection with high‐risk types of the human papillomavirus (HPV) has emerged as a major independent risk factor for head and neck carcinogenesis, particularly in oropharyngeal squamous cell carcinoma (OPSCC) [8]. The molecular pathogenesis of HPV‐mediated transformation is primarily driven by the viral oncoproteins E6 and E7, which orchestrate the degradation of critical tumor suppressor proteins p53 and pRb, respectively, thereby dismantling cellular safeguards against uncontrolled proliferation and promoting genomic instability [4, 9, 10].

### 1.3 The Anatomical Dichotomy and Controversial Role of HPV in Oral Carcinogenesis

While the etiological role of HPV in OPSCC is firmly established, its biological significance in oral squamous cell carcinoma (OSCC) remains controversial and represents an active area of investigation [8, 11]. Epidemiological studies report a highly variable prevalence of HPV in OSCC, with rates ranging from 10% to 25%, and with HPV16 being the predominant genotype detected [11]. The clinical implications of HPV infection in OSCC present a paradoxical scenario: several studies have associated HPV positivity with improved survival outcomes [12], while others report either no significant association or a link to a more aggressive disease course and poorer prognosis. This apparent contradiction highlights the complex and likely distinct interplay between viral infection, anatomical microenvironment, and tumor biology in the oral cavity compared to the oropharynx.

### 1.4 Cancer Stem Cells: The Architects of Therapeutic Resistance and Tumor Progression

A fundamental paradigm in cancer biology involves the recognition that tumor heterogeneity, aggression, and treatment failure are driven by a subpopulation of cells with stem‐like properties, the cancer stem cells (CSCs) [13]. These cells possess enhanced self‐renewal capacity, differentiation potential, and are primarily responsible for metastasis and resistance to conventional chemo‐ and radiotherapies [14, 15]. In HNSCC, CSCs can be identified and isolated based on the expression of specific cell surface markers, including CD44 and CD98, as well as high enzymatic activity of aldehyde dehydrogenase‐1 (ALDH1) [16, 17]. Clinically, the abundance of these CSC markers correlates strongly with adverse pathological features and poor outcomes. For instance, CD44 expression is a robust indicator of radioresistance and has been consistently associated with poor prognosis [16, 18, 19]. Similarly, ALDH1 activity enriches for tumorigenic cells, drives metastasis, and has been specifically linked to lymph node metastasis and shorter survival in OSCC [20, 21, 22, 23]. These observations firmly position CSCs as critical mediators of disease recurrence and therapeutic resistance in HNSCC.

### 1.5 Hypothesizing a Link Between HPV Status and the CSC Phenotype

An emerging and compelling hypothesis suggests that the favorable prognosis and enhanced treatment response observed in HPV‐positive oropharyngeal carcinomas may be mechanistically explained by fundamental differences in CSC biology. Seminal work by Rietbergen et al. [19] demonstrated that HPV‐positive OPSCCs exhibit significantly lower immunohistochemical expression of the CSC markers CD44 and CD98 compared to HPV‐negative tumors. This suggests that viral etiology may be associated with a less stem‐like and therefore less aggressive tumor phenotype. Supporting this, Näsman et al. [24] found that absent/weak CD44 intensity combined with HPV‐positive status indicated a very high survival rate in OPSCC. However, this compelling correlation remains profoundly underexplored in the context of HPV‐associated oral tongue carcinomas. The relationship is complex and unclear; for example, while some evidence suggests ALDH1+ cells are less frequent in HPV‐negative OSCC [25], the overarching biological model derived from oropharynx studies would predict a *reduced* CSC population in HPV‐positive tumors. This significant knowledge gap is exacerbated by the high prevalence and distinct clinical behavior of tongue SCC.

### 1.6 Study Rationale and Specific Objectives

The intricate and poorly understood relationship between HPV infection and CSC phenotype represents a critical area of investigation that may elucidate the molecular determinants of prognosis and therapeutic response in tongue SCC. To address this knowledge gap, the present study aims to: (1) Determine the prevalence of high‐risk HPV infection using molecular detection methods in a well‐characterized cohort of tongue squamous cell carcinomas from the interior of São Paulo State, Brazil; (2) Evaluate the immunohistochemical expression profile of a panel of key CSC markers (CD44, CD98, and ALDH1); (3) Correlate HPV status with CSC marker expression patterns and with established clinicopathological prognostic indicators to identify potential interactions between viral infection and stemness phenotype. We hypothesize that HPV‐positive tongue SCCs will demonstrate a distinct CSC profile characterized by reduced expression of stemness markers (CD44, CD98, and ALDH1) compared to HPV‐negative tumors. Elucidating this relationship will provide crucial mechanistic insights into the biology of OSCC, potentially revealing novel prognostic biomarkers and paving the way for more personalized therapeutic strategies that target the CSC niche in this anatomically and clinically significant malignancy.

## 2 Methods

### 2.1 Study Design and Case Selection

A retrospective search was conducted for all OSCC cases (ICD‐10 C00‐C06) registered between January 2013 and December 2018 at the Hospital Amaral Carvalho (HAC), Jaú, SP, Brazil. The study was approved by the National Research Ethics Committee (CONEP/CAAE: 88903617.0.3001.5434). From a total of 1047 cases, 452 involved the tongue (C01‐C02). A sample size calculation (95% confidence level, 10% margin of error) was performed, and 83 formalin‐fixed paraffin‐embedded (FFPE) specimens of invasive tongue SCC were randomly selected for analysis. Inclusion criteria mandated adult patients of both sexes with histopathologic confirmed invasive carcinoma and no prior history of chemotherapy or radiotherapy.

### 2.2 DNA Extraction and HPV Detection

DNA was extracted from 10 sections (4 μm) of each FFPE block using the QIAamp DNA FFPE Tissue Kit (Qiagen). DNA concentration and purity were measured spectrophotometrically (NanoDrop 2000). qPCR was performed on a ViiA 7 System (Applied Biosystems) using the qPCR GreenMaster lowROX (Cellco Biotec). Samples were first screened with generic HPV SOE primers (184 bp). Beta‐actin was amplified as an endogenous control. HPV‐positive samples were subsequently typed with specific primers for HPV6 (118 bp), HPV11 (120 bp), HPV16 (137 bp), and HPV18 (121 bp). Detailed primer sequences, cycling conditions, and other relevant PCR parameters are provided in (Supplementary Table S1).

### 2.3 Immunohistochemistry (IHC)

Sections (3 μm) were subjected to antigen retrieval in Tris/EDTA (pH 9.0). After blocking, slides were incubated overnight at 4°C with primary antibodies: anti‐CD44 (clone 156‐3C11, 1:400, Thermo Fisher Scientific), anti‐CD98 (clones MEM‐108, 1:100, Thermo Fisher Scientific & EPR3548(2), 1:50, Abcam), and anti‐ALDH1 (clone 44, 1:600, BD Transduction Laboratories). The reaction was revealed using the Dako EnVision + Dual Link System‐HRP and DAB chromogen. Appropriate positive and negative controls were used (Supplementary Figure S1).

### 2.4 Digital Image and Statistical Analysis

Digital images of the tumor invasion front were captured (Axiocam 234 HRc, Carl Zeiss), and quantitative analysis of IHC staining was performed using ImageJ software with IHC Profiler plugin, which automatically categorized the percentage of positivity into four scores: Negative, Low Positive (Low), Positive, and High Positive (High). Statistical analyses were conducted using GraphPad Prism 7.04. Survival was analyzed by the Kaplan–Meier method. The Kruskal‐Wallis test with Dunn's post‐test was used to compare IHC scores. Associations between clinicopathological features and marker expression were assessed by Chi‐square test. Correlations between variables were determined by Spearman's rank correlation coefficient. A *p*‐value < 0.05 was considered significant.

## 3 Results

### 3.1 Demographic and Clinicopathological Data

The initial cohort of 452 tongue SCCs was predominantly male (84.6%) and most frequent in patients aged 50–59 years (34.0%). Most presented with advanced disease (Stage IVa: 36.6%). The randomly selected subset of 83 cases used for molecular and IHC analysis also showed a male predominance (67.5%) and a peak incidence in the 50–59 age group (32.5%). However, in this subset, most cases were Stage II (38.6%) and moderately differentiated (G2, 49.4%) (Table 1). Kaplan–Meier analysis of the overall tongue SCC cohort showed a steep decline in survival for Stage IV patients, with none surviving beyond 18 months.

**TABLE 1:** Demographic and clinicopathological characteristics of tongue squamous cell carcinoma specimens evaluated for HPV status and cancer stem cell markers (*N* = 83).

Category	Subcategory	N	%
Age (years)	20–29	1	1.20
	30–39	5	6.02
	40–49	8	9.64
	50–59	27	32.53
	60–69	24	28.92
	70–79	14	16.87
	80–89	4	4.82
	90–95	0	0
Sex	Male	56	67.47
	Female	27	32.53
Clinical stage	Stage X (0)	1	1.20
	Stage I	25	30.12
	Stage II	32	38.55
	Stage III	17	20.48
	Stage IVa	7	8.43
	Stage IVb	0	0
	Stage IVc	1	1.20
Tumor grade	G1 Well‐differentiated	24	28.92
	G2 Moderately‐differentiated	41	49.40
	G3 Poorly‐differentiated	18	21.69

### 3.2 HPV Detection

All samples were positive for the beta‐actin control, confirming DNA quality. HPV DNA was detected in 2 of 83 cases (2.4%). One case was identified as HPV16‐positive (cq 27.31, Tm 77.03°C). The other case showed a mixed infection profile, with amplification for both HPV6 (cq 37, Tm 77°C) and HPV18 (cq 34, Tm 84°C). Both HPV‐positive patients were non‐smokers and non‐drinkers.

### 3.3 Cancer Stem Cell Marker Expression Profile

Quantitative analysis of immunohistochemical (IHC) staining revealed distinct expression profiles for the two CSC markers. CD44 exhibited intense and frequent membrane staining in the majority of specimens. Statistical analysis of the IHC scores revealed a highly significant difference between expression categories (Kruskal‐Wallis, *p* < 0.0001). Dunn's multiple comparisons test delineated a clear hierarchy: the expression levels in the CD44‐High and CD44‐Positive categories were not significantly different from each other (*p* > 0.9999), but both were significantly higher than those in the CD44‐Low (*p* < 0.0001) and CD44‐Negative (*p* < 0.0001) categories. Furthermore, CD44‐Low expression was significantly higher than CD44‐Negative (*p* < 0.0001) (Figures 1 and 3; Table 2).

**FIGURE 1:**
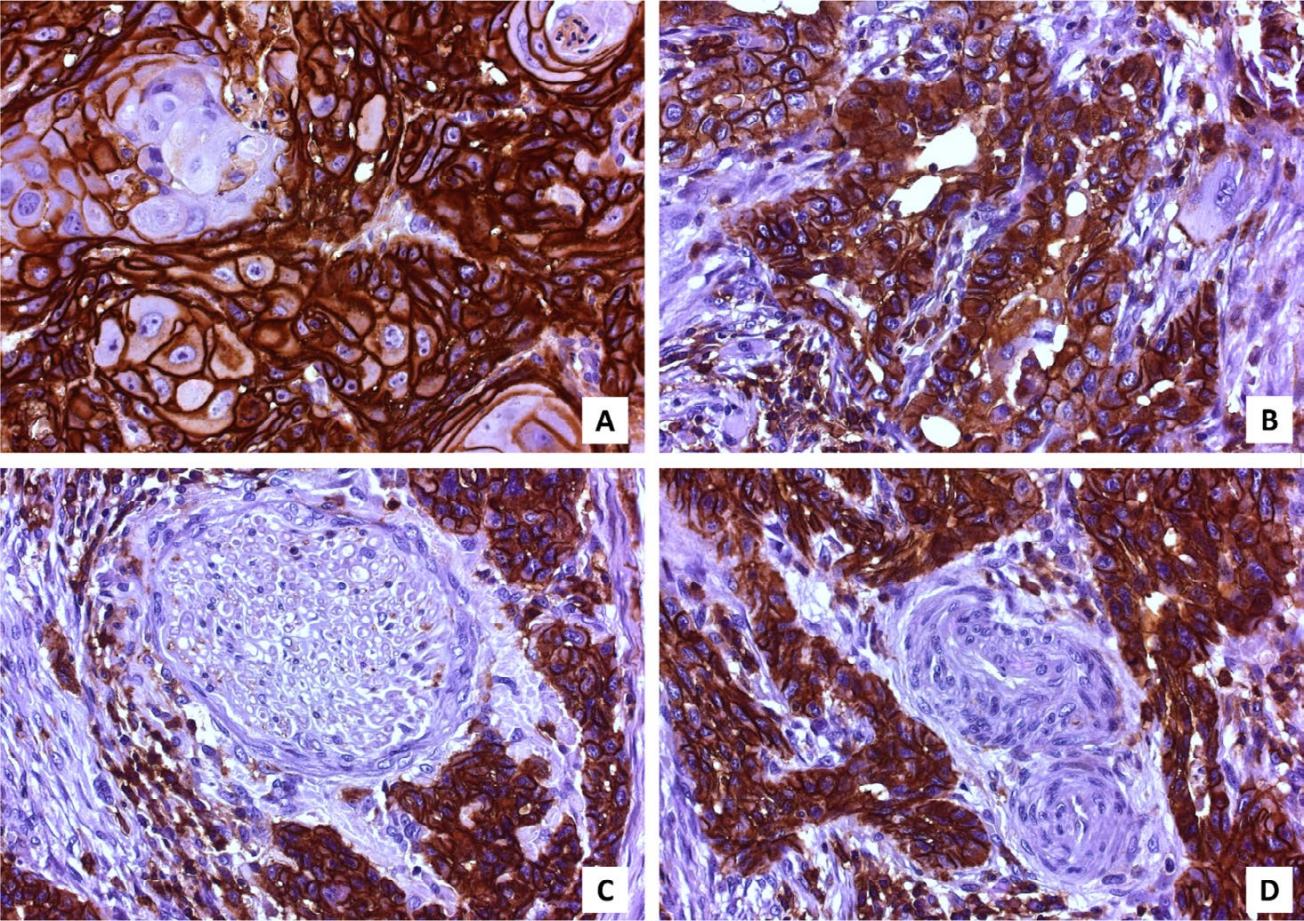
Immunohistochemical detection of CD44 in tongue SCC. (A–D) Representative photomicrographs of CD44 expression showing intense membrane staining in invasive tumor islands (A, B), perineural (C), and perivascular (D) invasion. (Original magnification: 400×).

**FIGURE 2:**
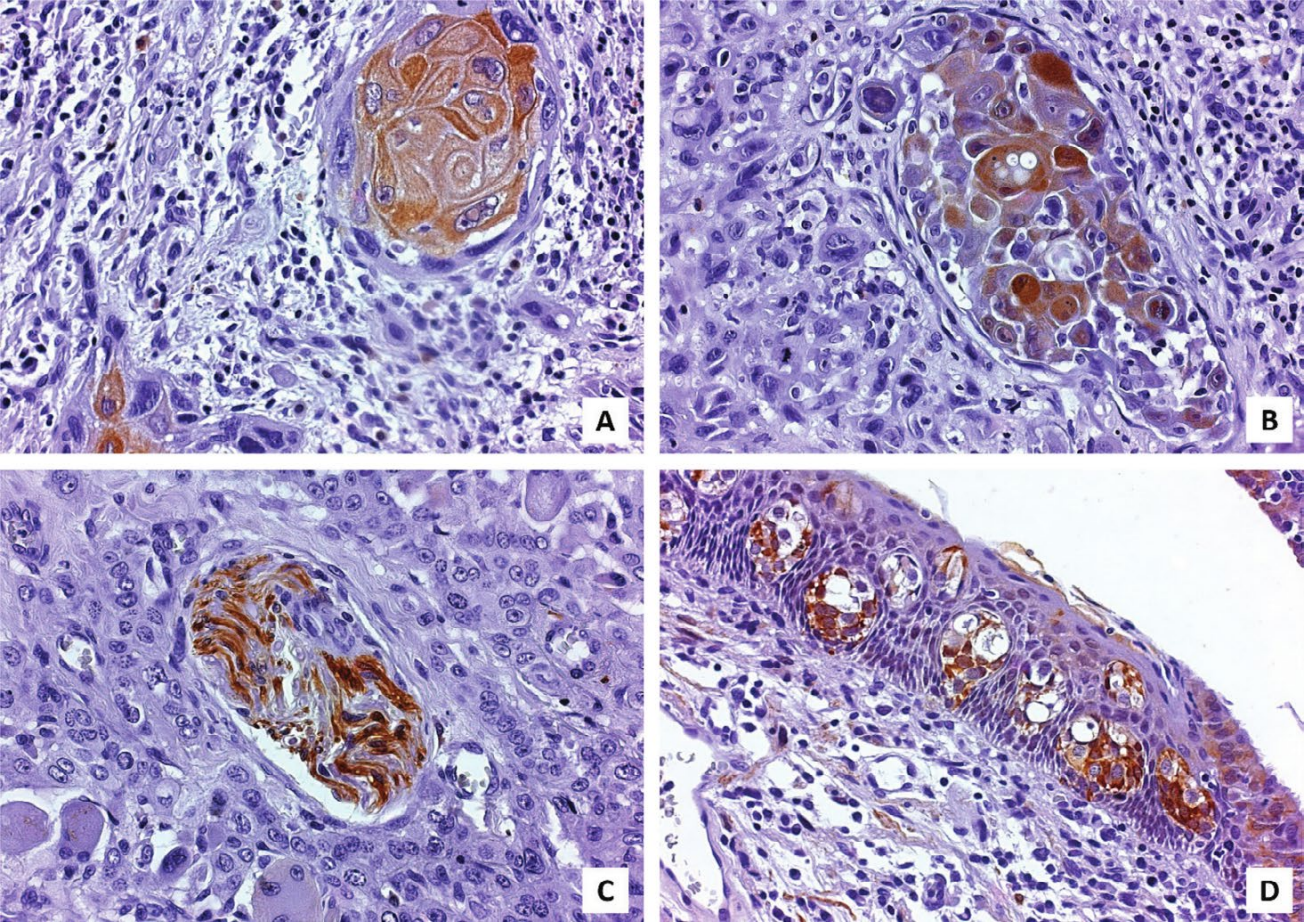
Immunohistochemical detection of ALDH1 in tongue SCC. (A–D) Representative photomicrographs of ALDH1 expression showing focal cytoplasmic staining in tumor islands (A, B) and in neural structures (C, D). (Original magnification: 400×).

In contrast, ALDH1 demonstrated a focal staining pattern, primarily restricted to neoplastic islands in the advancing tumor front. The overall difference between ALDH1 IHC score categories was significant (Kruskal‐Wallis, *p* < 0.0001). Post hoc analysis showed that the ALDH1‐High score was significantly less frequent than both the ALDH1‐Positive (*p* = 0.0099) and ALDH1‐Low (*p* = 0.0002) scores. The ALDH1‐High score was not statistically different from the ALDH‐1Negative category (*p* = 0.0703). No significant differences were observed between the ALDH1‐Positive, ALDH1‐Low, and ALDH1‐negative groups when compared to each other (all *p* > 0.9999) (Figures 2 and 3; Table 2). The marker CD98 was not detectable in any sample or positive control despite testing multiple antibodies and protocols, precluding its analysis.

**FIGURE 3:**
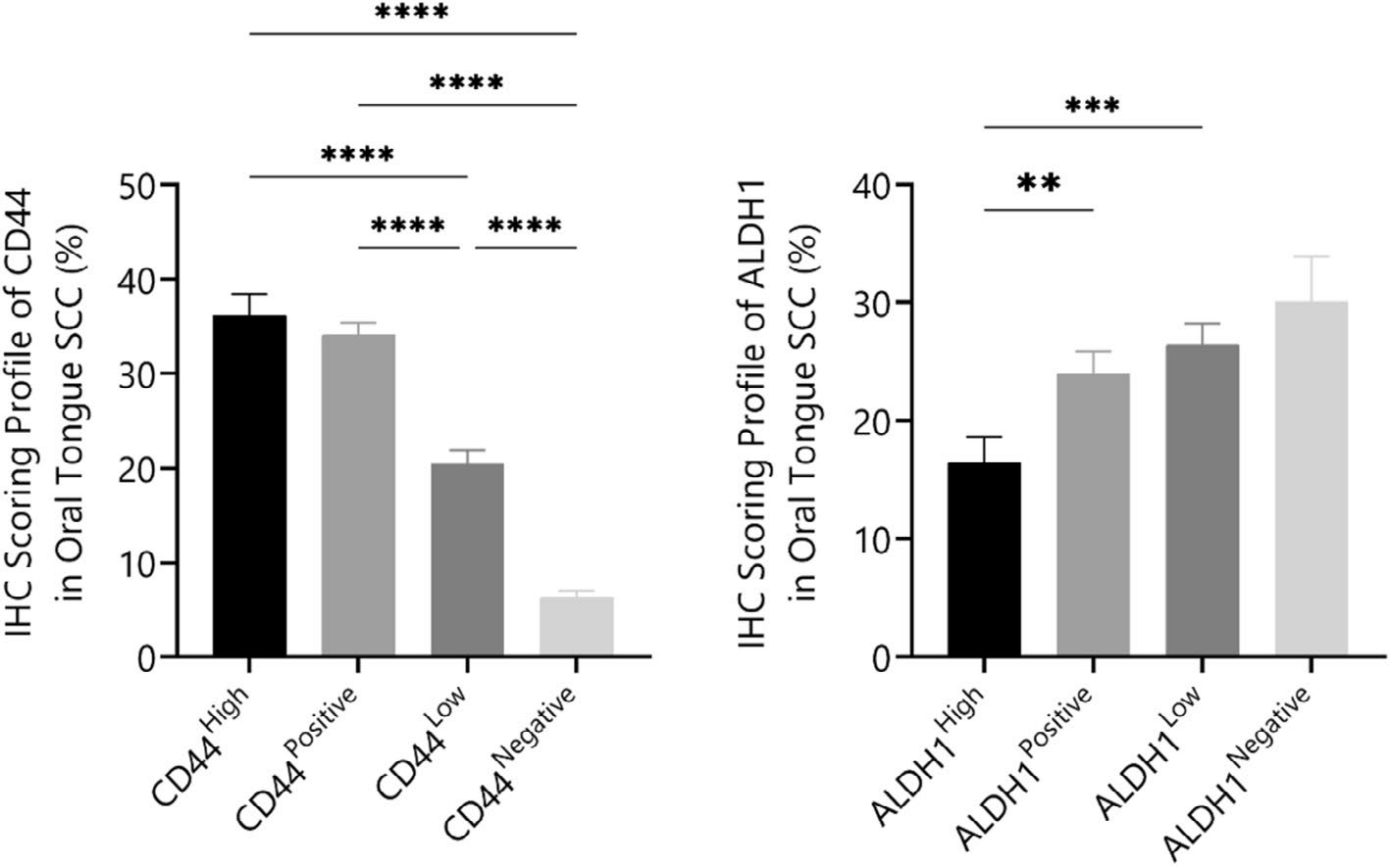
Quantitative analysis of IHC scores for CD44 and ALDH1. Kruskal‐Wallis test with Dunn's post‐test. ****p < 0.0001, *p < 0.05.

### 3.4 Correlation Analyses

Spearman correlation analysis revealed a statistically significant inverse correlation between HPV status (absent = 0, present = 1) and clinical stage (Stage X = 0, I/II/III/IV = 1/2/3/4) (r = −0.2377, *p* = 0.0305). Regarding CSC markers, a significant positive correlation was found between ALDH1 expression score (0–3) and both clinical stage (*r* = 0.5062, *p* = 0.0007) and tumor grade (*r* = 0.3352, *p* = 0.0322). No significant correlations were found between CD44 expression and the other variables tested (HPV, ALDH1, stage, grade). Chi‐square tests showed no significant association between CD44 or ALDH1 expression categories and demographic or clinicopathological parameters (Age, Sex, Stage, Grade) (Table 2).

**TABLE 2:** Statistically significant Spearman correlation analyses between HPV status, cancer stem cell marker expression, and clinicopathological parameters in tongue squamous cell carcinoma.

Variable 1	Variable 2	Spearman r	*p*‐value	Correlation
HPV Status	Clinical Stage	−0.2377	0.0305	Inverse
ALDH1 IHC Score	Clinical Stage	0.5062	0.0007	Positive
ALDH1 IHC Score	Tumor Grade	0.3352	0.0322	Positive

*Note:* HPV Status: 0 = absent; 1 = present. ALDH1 IHC Score: 0 = Negative; 1 = Low; 2 = Positive; 3 = High. Clinical Stage: 0 = Stage X; 1, 2, 3, 4 = Stage I, II, III, IV.

## 4 Discussion

This study investigated the interplay between HPV infection, CSC marker expression, and clinicopathological parameters in a defined population of tongue SCCs from Brazil. Our key findings are: (1) a very low prevalence of HPV (2.4%); (2) a hierarchical expression profile of CSC markers, with ubiquitous high CD44 and a distinct, less frequent ALDH1‐High expression; (3) a significant inverse correlation between HPV presence and clinical stage; and (4) a significant positive correlation between ALDH1 expression and both advanced clinical stage and higher tumor grade.

The low detection rate of HPV in our tongue SCC cohort (2.4%) aligns with previous findings that clearly distinguish the oral cavity from the oropharynx, reporting true HPV‐driven prevalence in oral sites to be around 5% or less [9, 10, 11]. This unambiguous difference underscores that in our population, traditional risk factors like tobacco and alcohol almost certainly remain the predominant drivers of tongue SCC, a profile consistent with the demographic characteristics of our cohort [5]. However, it is important to explicitly acknowledge a limitation regarding our data. HPV DNA was detected in only 2 out of 83 cases. Consequently, the statistical power for correlation analyses involving HPV status is extremely limited in our sample. While the inverse correlation with clinical stage aligns with the recurring notion that HPV‐positive oral cancers may be associated with a less advanced disease stage at diagnosis [12, 26], our findings must be interpreted with caution, and larger cohort studies are needed to confirm these trends in this specific population.

The robust expression of CD44 in the vast majority of tumors, where ‐High and ‐Positive expression categories were statistically indistinguishable and dominant, confirms its role as a foundational marker in OSCC and is consistent with its association with CSC populations [16, 27]. However, its lack of correlation with stage or grade suggests it may be a pervasive feature of tongue SCC cells in this etiological context, rather than a specific marker of aggressive subclones. In clear contrast, the expression pattern of ALDH1 was highly specific. The ALDH1‐High phenotype was significantly less frequent than lower expression categories and was uniquely associated with adverse pathological features. Its focal expression in the advancing tumor front and its significant positive correlation with stage and grade are particularly noteworthy. This pattern suggests that ALDH1‐high cells are enriched in more aggressive, invasive regions of the tumor and are positively selected during disease progression [20]. Our results strongly support the growing evidence that ALDH1 is a marker of aggressive behavior in OSCC, associated with epithelial‐mesenchymal transition, invasion, and metastasis [21, 23].

Beyond their prognostic value in established carcinomas, the investigation of CSC markers, particularly ALDH1, may hold significant translational relevance in the risk stratification of oral potentially malignant disorders (OPMDs) [28]. Assessing the expression of these markers in lesions such as oral leukoplakia and oral epithelial dysplasia could serve as a valuable tool to identify high‐risk pre‐malignant tissues with a greater potential for malignant transformation into squamous cell carcinoma, guiding closer clinical monitoring and earlier interventions [29].

The finding that ALDH1, but not CD44, correlated with adverse pathological features suggests that within the broad CD44‐positive CSC pool, ALDH1 demarcates a rarer, more aggressive subpopulation responsible for tumor progression in this context [14, 22]. This proposes a refined model for our population: a CD44‐High background, indicative of a stem‐like phenotype, upon which ALDH1 upregulation defines a high‐risk clone. This CD44‐High/ALDH1‐High profile, concomitant with low HPV prevalence, potentially identifies a subgroup of tumors driven by traditional carcinogens with a high risk of progression. This profile warrants validation in larger cohorts. The inability to detect CD98, despite using validated antibodies and protocols, was unexpected but suggests that CD98 is not a prevalent or reliable CSC marker in human tongue SCC, highlighting the importance of validating preclinical markers in clinical samples [17].

## 5 Conclusion

The present study provides insights into the biological characteristics of tongue SCC in a Brazilian population. We found a low prevalence of HPV infection, reinforcing that the disease burden in this region is primarily driven by traditional risk factors [5, 10]. We identified a strong association between the ALDH1‐High CSC subpopulation and adverse clinicopathological features, reinforcing the importance of CSCs in tongue cancer progression [13, 30]. The distinct expression patterns of CD44 (ubiquitous) and ALDH1 (focal and aggressive) suggest a hierarchy within the CSC compartment [15, 16]. The immunohistochemical profile of CD44‐High/ALDH1‐High, alongside low HPV detection, may help identify tumors with a distinct and aggressive biological behavior, potentially informing future prognostic stratification and guiding targeted therapeutic strategies against this specific CSC subpopulation.

## Funding

This work was supported by São Paulo Research Foundation (FAPESP Grant no. 2017/23791‐4).

## Conflicts of Interest

The authors declare no conflicts of interest.

## Supporting information


**Figure S1: Supplementary** (A) Positive control for CD44: Normal palatine tonsil exhibiting strong membranous staining in the basal and parabasal layers of the squamous epithelium. (B) Positive control for CD98: Normal placenta showing membranous positivity in the trophoblastic cells. (C) Positive control for ALDH‐1: Normal testis displaying cytoplasmic staining in Leydig cells. (D) Negative control: Section processed without the primary antibody, demonstrating the absence of non‐specific staining. (Original magnification: 400×).

**Table S1: Supplementary** Primer sequences, PCR parameters, and cycling conditions used for HPV detection and genotyping.

## Data Availability

The data that support the findings of this study are available from the corresponding author upon reasonable request.
