# Supplementary material for: Correlation Between Prognostic Indicators, HPV Status, and Cancer Stem Cell Markers in a Population of Human Tongue Carcinomas
Source: J Oral Pathol Med. 2026 Aug 16;55(9):1065–72. doi: 10.1111/jop.70175 (PMC13635275; doi:10.1111/jop.70175)
Supplement: Supplementary file 1 [file JOP-55-1065-s001.docx]

**Supplementary Material**

**Supplementary Table S1.** Primer sequences, PCR parameters, and cycling conditions used for HPV detection and genotyping.

| **Target** | **Primer Name** | **Sequence (5' → 3')** | **Concentration** | **Amplicon Size** |
| --- | --- | --- | --- | --- |
| HPV 6 | HPV6L1F | TGGGGTAATCAACTGTTTGTTACTGTGGTA | 400 nM | 118 bp |
| HPV 6 | HPV6L1R | GCATGTACTCTTTATAATCAGAATTGGTGTATGTG | 400 nM | 118 bp |
| HPV 11 | HPV11L1F | CTGGGGAAACCACTTGTTTGTTACTGTG | 400 nM | 120 bp |
| HPV 11 | HPV11L1R | CGCATGTATTCCTTATAATCTGAATTAGTGTATGTA | 400 nM | 120 bp |
| HPV 16 | HPV16L1F | TTGTTGGGGTAACCAACTATTTGTTACTGTT | 400 nM | 137 bp |
| HPV 16 | HPV16L1R | CCTCCCCATGTCTGAGGTACTCCTTAAAG | 400 nM | 137 bp |
| HPV 18 | HP18L1F | GCATAATCAATTATTTGTTACTGTGGTAGATACCACT | 400 nM | 121 bp |
| HPV 18 | HP18L1R | GCTATACTGCTTAAATTTGGTAGCATCATATTGC | 400 nM | 121 bp |
| HPV SOE | HPV SOE F | ANANGCTGTGCAKGNNCTAAAACGAAG | 300 nM | 184 bp |
| HPV SOE | HPV SOE R | AGTTTCCACTTCAGTATTGCCATA | 300 nM | 184 bp |
| Beta-actin | B ACTIN F | ACA CAA CTG TGT TCA CTA GC | 100 nM | -- |
| Beta-actin | B ACTIN R | CAA CTT CAT CCA CGT TCA CC | 100 nM | -- |

**PCR Parameters & Cycling Conditions:**

Equipment: ViiA™ 7 Real-Time PCR System (Applied Biosystems)
Chemistry: GreenMaster lowROX qPCR Kit (Cellco Biotec)
Reaction Volume: 20 µL (18 µL master mix + 2 µL DNA at 25 ng/µL)
Detection Threshold: Automatically calculated by the ViiA™ 7 software baseline settings (or specify your custom threshold here if manually set).
**Cycling Stages (45 Cycles):**
1. UNG Treatment: 50 °C for 2 minutes
2. Initial Denaturation & Polymerase Activation: 95 °C for 10 minutes
3. PCR Cycles:
 - Denaturation: 95 °C for 10 seconds
 - Annealing: 55 °C for 10 seconds
 - Final Extension: 72 °C for 60 seconds (Fluorescence acquired)
4. Melting Curve Analysis:
 - 95 °C for 15 seconds
 - 60 °C for 1 minute
 - Dissociation stage: 95 °C for 15 seconds
