# Supplementary material for: Correlation Between Prognostic Indicators, HPV Status, and Cancer Stem Cell Markers in a Population of Human Tongue Carcinomas
Source: J Oral Pathol Med. 2026 Aug 16;55(9):1065–72. doi: 10.1111/jop.70175 (PMC13635275; doi:10.1111/jop.70175)
Supplement: Supplementary file 2 [file JOP-55-1065-s002.zip › jop70175-sup-0001-FigureS1.docx]

**Supplementary Figure S1**

**(A)** Positive control for CD44: Normal palatine tonsil exhibiting strong membranous staining in the basal and parabasal layers of the squamous epithelium. **(B)** Positive control for CD98: Normal placenta showing membranous positivity in the trophoblastic cells. **(C)** Positive control for ALDH-1: Normal testis displaying cytoplasmic staining in Leydig cells. **(D)** Negative control: Section processed without the primary antibody, demonstrating the absence of non-specific staining. (Original magnification: 400x).
